# A more significant role for insertion sequences in large-scale rearrangements in bacterial genomes

**DOI:** 10.1128/mbio.03052-24

**Published:** 2024-12-05

**Authors:** Wing Y. Ngan, Lavisha Parab, Frederic Bertels, Jenna Gallie

**Affiliations:** 1 Microbial Evolutionary Dynamics Group, Department of Theoretical Biology, Max Planck Institute for Evolutionary Biology, Plön, Germany; 2 Microbial Molecular Evolution Group, Department of Microbial Population Biology, Max Planck Institute for Evolutionary Biology, Plön, Germany; Massachusetts Institute of Technology, Cambridge, Massachusetts, USA

**Keywords:** insertion sequence, evolution, mobile genetic elements, *Pseudomonas fluorescens*, *Escherichia coli*, DNA repair, genomic rearrangement

## Abstract

Insertion sequences (ISs) are mobile pieces of DNA that are widespread in bacterial genomes. IS movements typically involve (i) excision of the IS element, (ii) cutting of target site DNA, and (iii) IS element insertion. This process generates a new copy of the IS element and a short duplication at the target site. It has been noted that, for some extant IS copies, no target site duplications (TSDs) are readily identifiable. TSD absence has been attributed to degeneration of the TSD after the insertion event, recombination between identical ISs, or adjacent deletions. Indeed, the latter two—recombination between ISs and adjacent deletions—are frequent causes for the absence of TSDs, which we demonstrate here in an analysis of genome sequence data from the Lenski long-term evolution experiment. Furthermore, we propose that some IS movements—namely, those that occur in association with large-scale genomic rearrangements—do not generate TSDs, and occur without evidence for recombination between ISs or adjacent deletions. In support of this hypothesis, we provide two direct, empirical observations of such IS transposition events: an IS*5* movement plus a large deletion in *Escherichia coli* C, and an IS*481* movement occurring with a large duplication in *Pseudomonas fluorescens* SBW25. Although unlikely, it is possible that the observed deletion and associated IS movement occurred in two successive events in one overnight culture. However, an IS at the center of a large-scale duplication is not readily explained, suggesting that IS element activity may promote both large-scale deletions and duplications.

**IMPORTANCE:**

Insertion sequences are the most common mobile genetic elements found in bacterial genomes, and hence they significantly impact bacterial evolution. We observe insertion sequence movement at the center of large-scale deletions and duplications that occurred during laboratory evolution experiments with *Escherichia coli* and *Pseudomonas fluorescens*, involving three distinct types of transposase. We raise the possibility that the transposase does not mediate DNA cleavage but instead inserts into existing DNA breaks. Our research highlights the importance of insertion sequences for the generation of large-scale genomic rearrangements and raises questions concerning the mechanistic basis of these mutations.

## INTRODUCTION

Genomic rearrangements, such as large duplications and deletions, are among the most common types of mutation in bacteria (1–3). Such rearrangements occur as a result of recombination between distant genomic regions. In bacterial genomes, dispersed copies of mobile genetic elements—such as insertion sequence (IS) and repetitive extragenic palindromic (REP) sequences—frequently act as hot spots for such recombination events (4, 5). In the case of ISs, genomic rearrangement is thought to be promoted in at least two ways: (i) by the provision of homologous stretches of DNA that serve as the raw material for recombination, and (ii) through the enzymatic activity encoded within the IS element (6).

IS elements, of which there are many families, are the smallest autonomous mobile genetic elements in bacterial genomes (7). Ranging in size from ~0.7 kb to ~2.5 kb, IS elements typically consist of a transposase gene flanked by two, usually inverted, repeats. Most IS elements move and replicate by binding to their terminal repeats and cutting themselves out of the DNA. The transposase-DNA complex is then inserted elsewhere in the genome, sometimes at specific target sites. The entire transposition process occurs during DNA replication, ensuring that both the original and new IS copies are maintained in the mother and daughter genomes (through DNA repair mechanisms) (8–10). During insertion, most IS elements generate a target site duplication (TSD; sometimes called “direct target repeats”) of between 1 bp and 14 bp, depending on the IS family (10). These TSDs occur because the transposase cuts the target DNA at different positions on the leading and lagging strands. The resulting overhangs are repaired after the IS element is pasted into the genome, leaving a short, direct repeat at the insertion site.

Through repeated transposition activity, bacterial genomes may carry numerous (nearly) identical, dispersed copies of a particular IS element. Recombination between dispersed IS elements is a common cause of large genomic rearrangements. One example is provided by Lenski’s long-term evolution experiment (LTEE), in which 12 populations of *Escherichia coli* B have been evolving for many thousands of generations (11, 12). In one evolving population (Ara-1), five of the nine (~55%) identified large genomic rearrangements are due to recombination between dispersed IS copies (5). Similarly, in an experiment with *Salmonella enterica* (Typhimurium), of 1,800 spontaneous duplication mutants trapped on a plasmid, 97% arose by recombination between flanking IS elements (13). Intriguingly, when the enzymatic activity of these IS elements was deactivated—either by replacement with two copies of a gene of identical length or by changing the start codon of the transposase gene—the duplication frequency was reduced by two-thirds (13). This result suggests that, in addition to providing the raw material for homologous recombination, transposase activity itself increases recombination rates and hence intra-genomic rearrangement frequencies.

One of the most common large-scale genomic rearrangements caused by ISs is adjacent deletions (14). An adjacent deletion occurs when the transposase excises not only the transposon but also adjacent DNA sequences. During this process, a cointegrated structure can form that contains both the IS and the captured flanking DNA. The resolution of the cointegrate usually leads to the maintenance of the IS but the loss of the flanking DNA (15), sometimes resulting in large-scale deletions adjacent to an existing IS.

In this work, we have observed and analyzed large-scale rearrangements caused (or promoted) by the activity of three distinct ISs: (i) IS*150* from *E. coli* B REL606 is part of the IS*3* family and the IS*150* group, (ii) IS*5* from *E. coli* C is part of the IS*5* family and the IS*903* sub-group, and (iii) IS*481* from *P. fluorescens* SBW25 is distantly related to the IS*3* family. The transposition mechanisms for members of the IS*3* and IS*5* families are well described, as outlined below.

IS elements from the IS*3* family (including IS*150*) mainly transpose via donor primed replicative transposition (16, 17). Hence, IS*3* family members generally transpose via a copy-and-paste mechanism rather than a cut-and-paste mechanism. IS*3* family members usually display two open reading frames (*orfA* and *orfB*), from which three proteins (OrfA, OrfB, and OrfAB) are produced as a result of programmed frameshifting (18). The OrfAB fusion protein is required for the successful transposition of IS*3* family members. Interestingly, the artificial fusion of *orfA* and *orfB* leads to an increased rate of adjacent deletions (19).

IS elements from the IS*5* family transpose through replicative and conservative transposition (20). Of these, the transposition mechanism of IS*903* transposases has been described in particular detail. Adjacent deletions are common in IS*903*; these require both ends of the IS and are thought to depend on the formation of a cointegrate. The involvement of the cointegrate, which is a hallmark of replicative transposition in IS*5* and IS*3*, suggests that the replicative transposition pathway is required for adjacent deletions (15).

Here, we present observations that support an active role for IS elements during the formation of genomic rearrangements. We demonstrate and characterize the insertion of new IS element copies in association with (i) large deletions in *E. coli* B REL606, (ii) a large deletion in *E. coli* C, and (iii) a large duplication in *Pseudomonas fluorescens* SBW25. While the rearrangements in *E. coli* B result either from recombination between identical IS elements or adjacent deletions, it is more difficult to attribute the large-scale deletion in *E. coli* C—and impossible to attribute the large-scale duplication in *P. fluorescens*—to either recombination between homologous sequences or adjacent deletions. These rearrangements are not mediated through the recombination of two IS copies. Instead, an IS element is found either separating the two copies of a duplicated DNA sequence or at the center of a DNA sequence deletion event. Interestingly, these IS element insertions do not leave TSDs, suggesting that the IS-encoded DNA cleavage function is not involved in this process. While the deletion event we observed could be explained through adjacent deletions, the occurrence of two consecutive mutational steps within a 24-hour window suggests that this particular mutation was generated by another mechanism. We propose that IS elements are capable of inserting into DNA strand breaks that occur during the resolution of intragenomic duplication or deletion events.

## RESULTS

### Observation 1: frequent IS-Del mutations occurring in the LTEE caused by adjacent deletions

**Fig 1:**
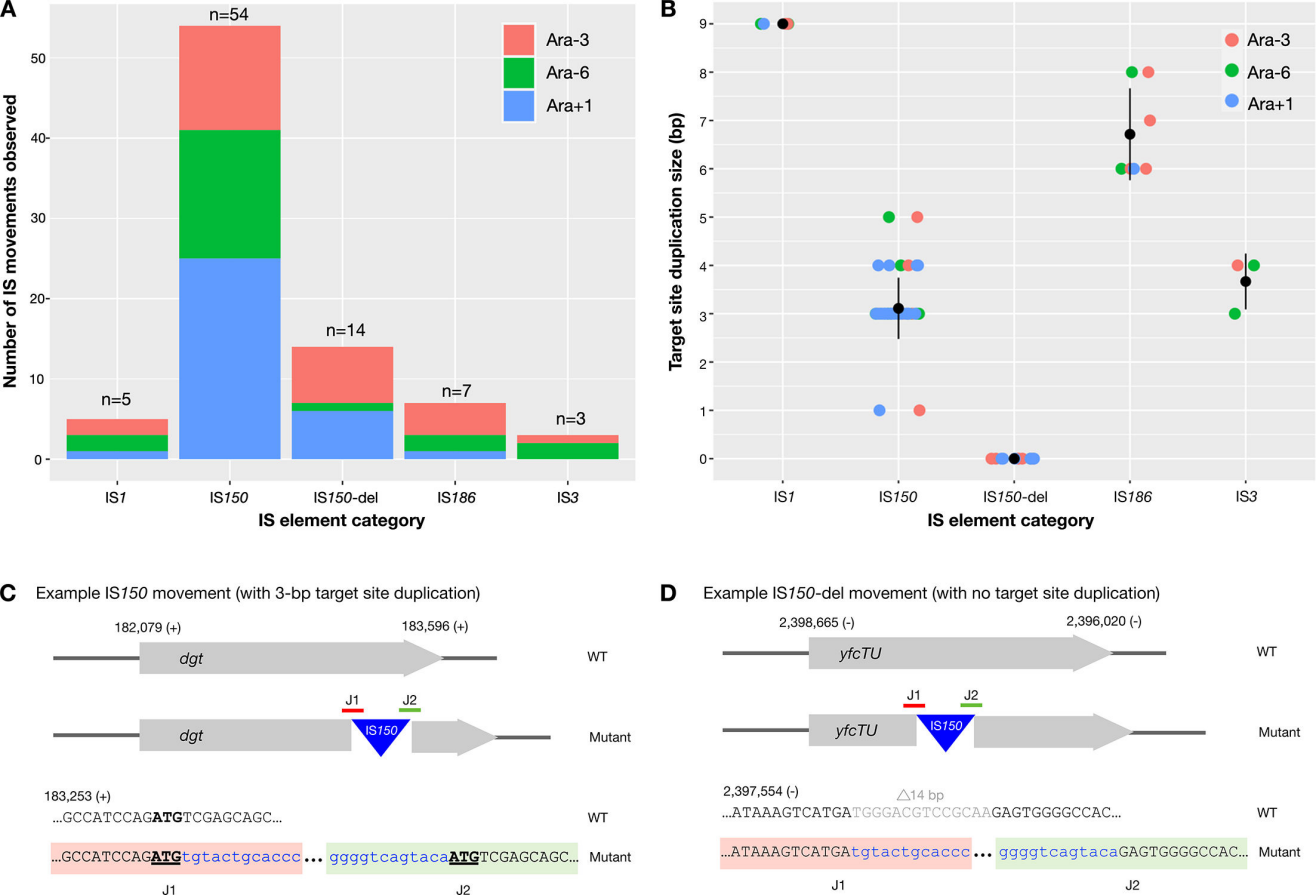
Characterization of IS transposition events in three LTEE populations at 60,000 generations (Ara+1, Ara-3, and Ara-6). (**A**) Eighty-three distinct IS transposition events were identified, involving IS elements from four families (IS*1,* IS*150*, IS*186,* and IS*3*). In the case of the IS*150* family, the 68 transposition events occurred in two categories: those that are not associated with large deletions (54, category “IS*150*”), and those that are associated with large deletions (14 events, category “IS*150*-del”). (**B**) Across all four IS families, each of the 69 transposition events not associated with a large deletion generated a TSD (1 bp to 9 bp), while none of the 14 IS*150-*del transposition events generated TSDs. Colored circles indicate the length of the TSD per transposition event, and black circles + lines show the mean + standard deviation for each IS family. (**C**) An example of an IS*150* insertion event that is not associated with a deletion. The event was identified in Ara+1 and resulted in the insertion of a new IS*150* element in the *dgt* gene plus the generation of a 3 bp direct repeat at the target site (bold and underlined). Black text = *dgt*, blue text = IS*150*. (**D**) An example of an IS*150-*del insertion event that is associated with a 14 bp deletion. The event was identified in Ara+1 and resulted in the insertion of a new IS*150* element in the *yfcTU* gene, and the deletion of a 14 bp sequence from *yfcTU* (light gray text). No direct repeats are generated at the target site. Black/gray text = *yfcTU*, blue text = IS*150*.

In order to assess whether IS-rearrangement mutations without the involvement of TSDs occur in publicly available data, we turned to the *E. coli* B LTEE. The Illumina sequencing reads for three 60,000-generation populations with particularly high IS activity levels (namely, Ara+1, Ara-3, Ara-6) were analyzed with breseq (21). A total of 83 IS movement events were predicted, encompassing four IS families (in decreasing order of prevalence: IS*150* [IS*3* family] [68]*,* IS*186* [IS*4* family] [7], IS*1* [5], IS*3* [3]) (Fig. 1A; Table S1). Of these, 14 (~17%) occurred in association with a large deletion (of between 14 bp and 48,894 bp). All 14 simultaneous IS and deletion events involve IS*150* family elements.

Having established that two types of IS movements (i.e., those that occur alone, and those that occur together with a genomic rearrangement) exist, we next looked at whether these two transposition categories are likely to occur via distinct molecular mechanisms. To begin, we investigated the identifiable TSDs in each of the IS movement events. As outlined in the introduction, most IS transposition events generate a scar at the target site (short duplications of ~1 bp to 14 bp). Such scars are due to the transposon enzymatically cutting the target DNA in different positions on the leading and lagging strands during the transposition event. In line with this, all 69 standard IS movements—those that do not coincide with a deletion—observed here generated a TSD of between 1 bp and 9 bp (Fig. 1B and C; Table S1). Contrastingly, none of the 14 IS movements associated with a large deletion generated a TSD of any size (Fig. 1B through D).

Next, we analyzed whether the large deletions occurred as part of a two-step process (i.e., whether an insertion of the IS element preceded the deletion event). If an insertion of an IS*150* element preceded the deletion, then it is likely that the deletion is adjacent. An adjacent deletion can occur as part of the cointegrate formation process (see the introduction). For all 14 IS-associated deletion mutations, we identified an IS element present at the same position in the genome at an earlier time point of the LTEE. Often, the adjacent deletion was small at first and increased in size over time. Hence, all 14 IS-del mutations can be explained as multistep mutations: (i) insertion of an IS element, (ii) occurrence of an adjacent deletion next to the IS element, and (sometimes) (iii) extension of the deletion.

### Observation 2: IS activity associated with a large deletion event in *E. coli* C

**Fig 2:**
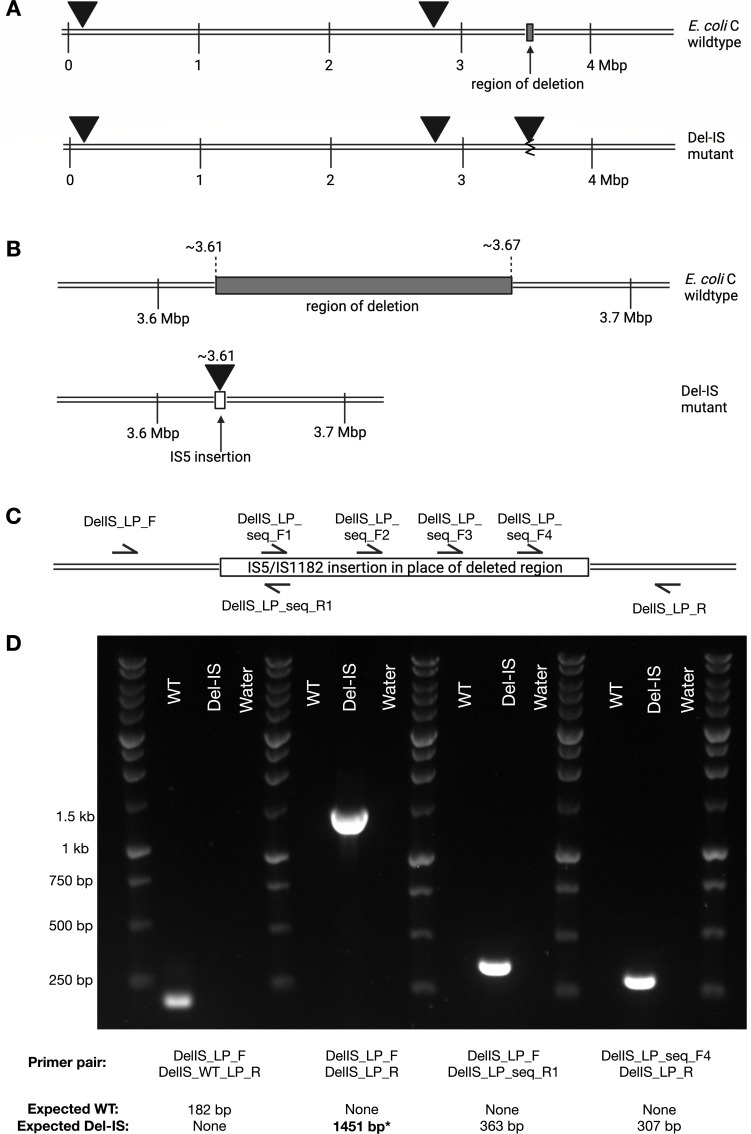
Ec-Del-IS carries a genomic rearrangement consisting of a large deletion and an IS*5* transposition event. (**A**) A whole-genome view. Linear representations of *E. coli* C wild type (top) and the derived Ec-Del-IS (bottom) (cut at the origin of replication, *ori*). The wild-type genome contains two identical copies of the 1,051 bp IS*5* sequence (*B6N50_00555, B6N50_14295*; solid triangles). The IS mutant contains an ~62 kb deletion (open rectangle; top) and an extra copy of the IS element (third solid triangle; bottom). (**B**) A close-up view showing the 62,007 bp deletion. (**C**) Cartoon showing positions of the PCR and sequencing primers used to verify the mutation. (**D**) PCR-mediated amplifications of the rearrangement locus in Ec-Del-IS (versus wild type). In order to confirm the sequence across the genomic rearrangement, the starred 1,451 bp PCR product (which includes the new IS copy and the emergent DNA junctions Del1-IS1 and IS2-Del2) was Sanger sequenced with the seven primers shown in panel C.

During an evolution experiment with *E. coli* C and phage ΦX174, movement of an IS*5* (sub-group IS*903*) element was observed in association with a large deletion (22). Here, we provide an in-depth computational and empirical analysis of this genomic rearrangement. The strain of interest, Ec-Del-IS, contains a 62,007 bp deletion (genomic positions 3612369–3674375) and an insertion of a new IS*5* element copy at the deletion site (see Text S1; Table S2). The *E. coli* C IS*5* element is ~1 kb in length, almost the entirety of which is a 918 bp transpose gene flanked by 14 bp inverted repeats, without any obvious TSD. Two identical, full copies of the element are present in the wild-type *E. coli* C genome (transposase gene locus tags *B6N50_00555* and *B6N50_14295*), and three are found in the Ec-Del-IS genome (Fig. 2A and B).

The emergence of the deletion-IS mutation in Ec-Del-IS was demonstrated using PCR (Fig. 2C and D), and the sequence of the mutated region was verified by Sanger sequencing. No TSDs are observable at the insertion site, suggesting that the IS element insertion occurred at the same time as the deletion event (i.e., with the IS*5* element inserted into the double-stranded DNA break).

### Observation 3: IS activity associated with a large duplication event in *P. fluorescens* SBW25

#### SBW25-Dup-IS carries a new IS element separating two copies of a duplicated DNA sequence

**Fig 3:**
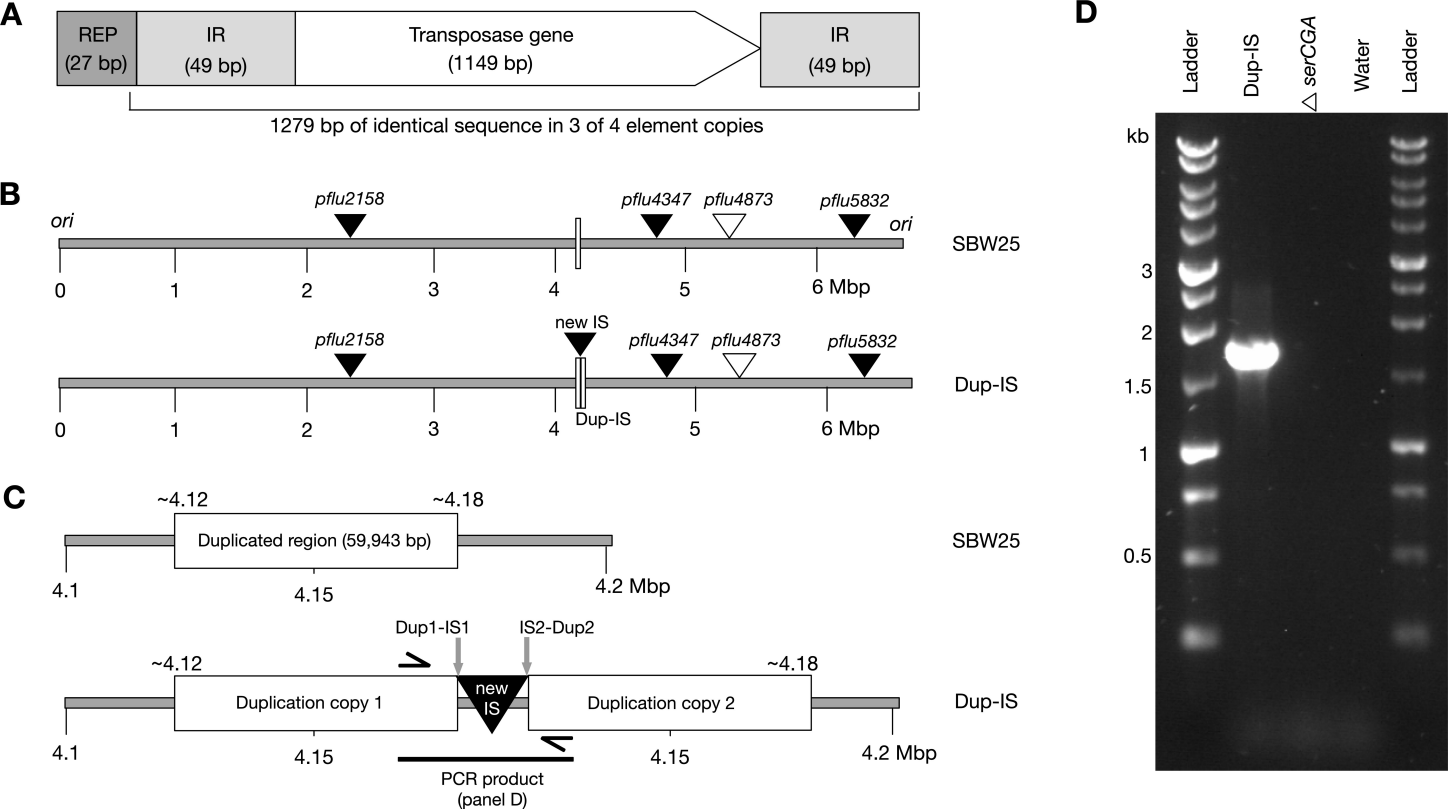
SBW25-Dup-IS carries a genomic rearrangement consisting of a large duplication and an IS*481* transposition event. (**A**) Conserved structure of the SBW25 IS*481-*family transposable element (IR = inverted repeat). (**B**) Linear representations of the SBW25 (top) and SBW25-Dup-IS mutant (bottom) genomes (cut at the origin of replication, *ori*). SBW25 contains three IS*481* copies in which the flanking IRs and transposase genes (*pflu2158*, *pflu4347*, *pflu5832*; solid triangles) are identical in sequence, and a fourth copy carrying eight SNPs and a 1 bp deletion in the transposase gene (*pflu4873*; open triangle). The 1 bp deletion is predicted to cause truncation of the transposase protein (R347G*), presumably rendering Pflu4873 enzymatically inactive. SBW25-Dup-IS contains a new, fifth IS copy (solid triangle) and a large duplication (open rectangles). (**C**) Cartoon enlargement of the rearrangement locus. In SBW25-Dup-IS, the two 59,943 bp duplication copies are separated by the new 1,279 bp IS*481* copy (giving a total of 61,222 bp extra DNA). Black, one-sided arrows indicate the approximate placement of the primers used for the PCR in panel D. (**D**) PCR-mediated amplification of the rearrangement locus in SBW25-Dup-IS. The 1,746 bp PCR product includes the new IS copy and the two emergent, flanking DNA junctions (Dup1-IS1 and IS2-Dup2).

The *P. fluorescens* SBW25 genome contains four dispersed copies of an ~1.3 kb IS*481*-family element (Fig. 3A and B). Each copy consists of (i) a transposase gene flanked on either side by 49 bp inverted repeats, and (ii) an upstream REP sequence. In three copies, the transposase gene and inverted repeats are identical in sequence, while the remaining copy differs at nine positions within the transposase gene. Three of the four IS*481* copies are encoded on the lagging strand and one (*pflu5832*) on the leading strand. The presence of these multiple highly conserved copies strongly implies (relatively) recent transposition activity. However, to the best of our knowledge, there have been no direct observations of this—or any other—IS element moving within SBW25 populations. Here, we observe an IS*481* transposition event in SBW25.

The strain of interest, SBW25-Dup-IS, was isolated from a laboratory population founded by a slow-growing strain (tRNA gene deletion mutant SBW25Δ*serCGA* [23]). Whole-genome re-sequencing revealed that, in line with previous strains isolated under similar conditions (23, 24), SBW25-Dup-IS contains a large, intragenomic duplication: a 59,943 bp duplication of genomic positions 4119903–4179845. However, the SBW25-Dup-IS duplication differs from prior reports in that the two copies of the duplicated region are separated by a new, fifth copy of the IS*481* element (Fig. 3B and C; Table S3). This proposed Dup1-IS-Dup2 genomic arrangement was confirmed in the laboratory by PCR-mediated amplification and Sanger sequencing of the emergent DNA fragment—encompassing the new IS*481* copy and the novel flanking regions—from SBW25-Dup-IS (Fig. 3D; Text S2).

**Fig 4:**
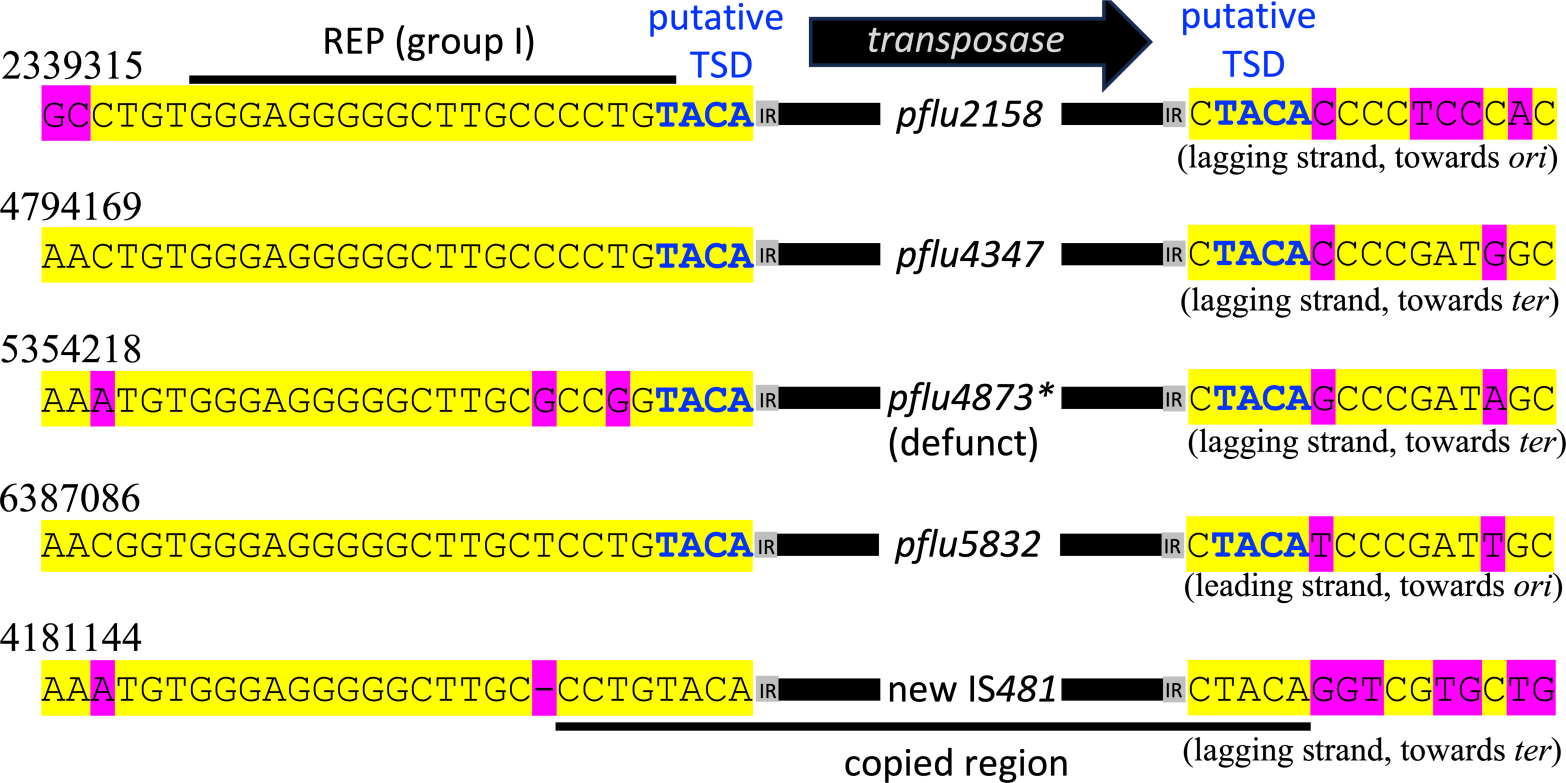
Insertion sites of four established, and one emergent, IS*481* element in the SBW25-Dup-IS genome. The upstream sequence of each IS*481* insertion site is highly conserved (left; 5' end of IS). IR = inverted repeat; yellow highlighting = consensus; pink highlighting = deviates from consensus; bold blue text = putative TSDs (note that in the new IS*481* copy, no putative TSD exists; see text). In each case, the IS*481* sequence has been inserted into the same position of a REP sequence, thereby either (i) disrupting a REPIN (*pflu4347*, *pflu4873, pflu5832*), (ii) inserting into a REP sequence (*pflu2158*), and/or (iii) inserting into a REP tandem repeat (new IS*481* insertion) (26, 27).

Elucidation of the precise Dup-IS-Dup nucleotide sequence revealed two points of interest. Firstly, the Dup-IS-Dup sequence does not display the 4 bp TSD that is observed for the four other SBW25 IS*481* elements. Indeed, no TSD of any size was observed; notably, the 4 bp sequence that we previously mistook for the TSD was copied along with the rest of the insertion sequence, rather than generated during the downstream insertion event (see Fig. 4). Secondly, insertion occurred into a REP sequence. Analysis of the upstream region of the four previously-existing IS*481* copies reveals a REP sequence at the 5' end of each, suggesting that REPs—of which there are hundreds of copies in the SBW25 genome (25)—can act as IS*481* target sites (Fig. 4). The REP sequence associated with the new IS copy differs from the others in that it lies within a cluster of 12 tandemly-repeated Group 1 and Group 3 REP sequences. There are 11 similar clusters of 4–12 tandemly repeated Group 1 and Group 3 REP sequences dispersed around the SBW25 chromosome (26, 27). It has previously been hypothesized (26, 27) and empirically observed (23, 24) that these REP clusters can serve as recombination hot spots, resulting in large-scale genomic rearrangements.

The work in this section characterizes a complex genomic rearrangement event in SBW25-Dup-IS, consisting of (i) the duplication of ~60 kb genomic DNA, and (ii) the insertion of a new IS*481* copy at the junction of the two duplication fragment copies. The endpoints of the new Dup-IS-Dup genomic arrangement suggest that the duplication event was originally promoted by a tandem REP cluster, but before the duplication could be fully resolved, the IS*481* element was inserted into the double-stranded DNA break.

#### The new IS copy, and associated duplication, is readily lost

Above, we demonstrated the emergence of a new, fifth copy of the SBW25 IS*481* element in conjunction with a large duplication. Given that large, tandem duplications are readily lost—usually without a trace—from bacterial genomes (3, 24, 28, 29), we wanted to investigate the fate of the new IS copy. That is, if the duplication is lost from SBW25-Dup-IS, does the new IS copy remain?

**Fig 5:**
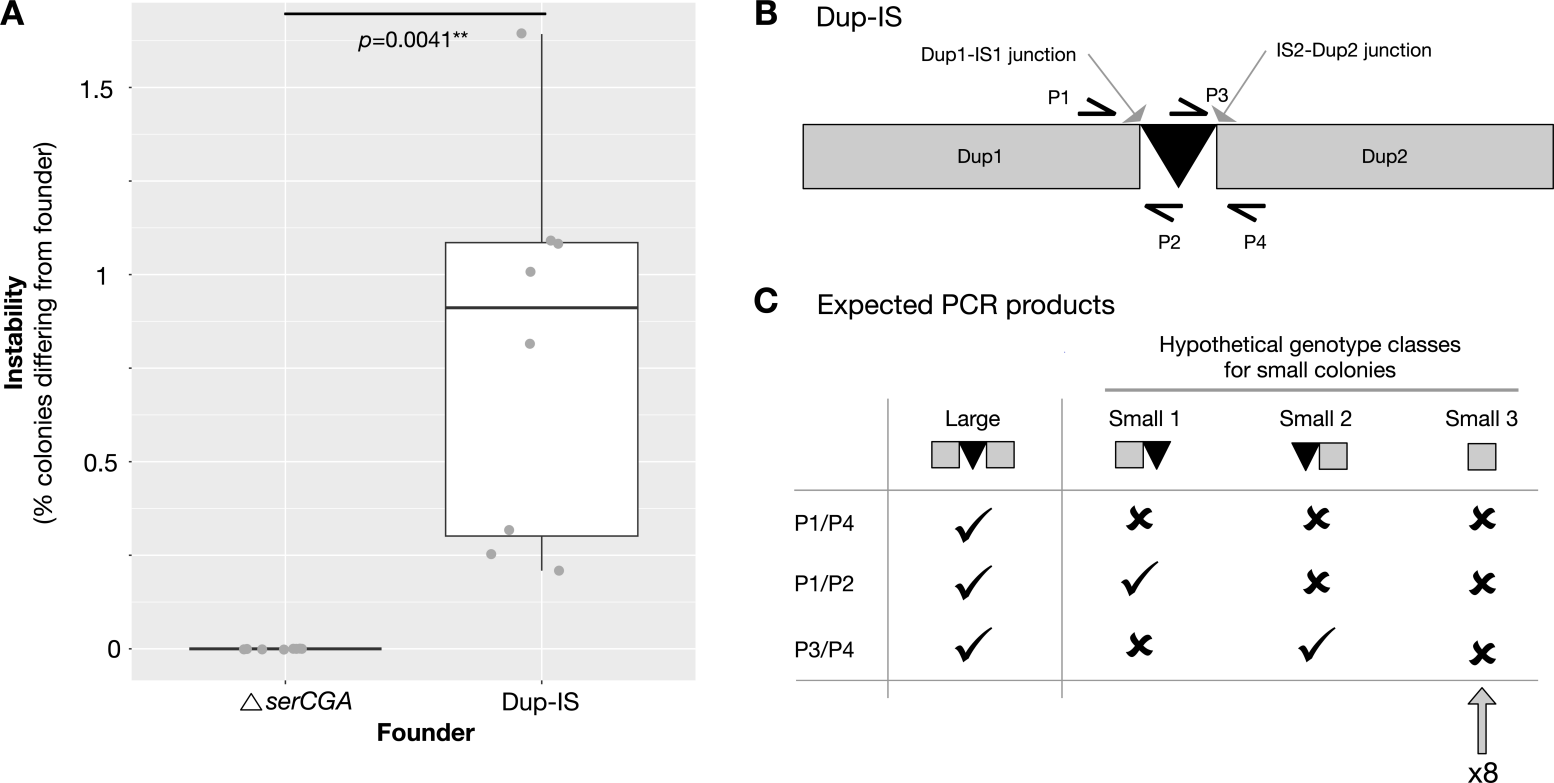
The new IS*481* copy is readily lost from SBW25-Dup-IS, along with the duplication fragment. (**A**) The stability of colony size in ancestral SBW25Δ*serCGA* versus derived strain SBW25-Dup-IS (~60 kb duplication fragment). Colonies plated from overnight cultures of SBW25Δ*serCGA* consistently retain the SBW25Δ*serCGA* phenotype, while those from SBW25-Dup-IS do not. Eight independent replicates were performed per strain (gray circles), with a minimum of 146 colonies counted per replicate. A Wilcoxon rank sum test was used to test for a difference in median instability (***P < 0.001, **P < 0.01, *P < 0.5). (**B**) Cartoon of SBW25-Dup-IS genotype showing the position of the two emergent junctions (Dup1-IS1 and IS2-Dup2), and the placement of primers to amplify each junction as follows: (i) primers P1/P4 to amplify both junctions and the new IS*481* copy in a single 1,746 bp product, (ii) primers P1/P2 to amplify Dup1-IS1 (359 bp product), and (iii) primers P3/P4 to amplify IS2-Dup2 (465 bp product). (**C**) Expected PCR products large colonies (i.e., SBW25-Dup-IS) and three hypothetical genotypic classes of SBW25-Dup-IS-derived small colonies: small-1 (retention of IS*481* and the Dup1-IS1 junction), small-2 (retention of IS*481* and the IS2-Dup2 junction), and small-3 (loss of IS*481* and both junctions). All eight small colony isolates show the PCR pattern expected for small-3 (see Fig. S1 for PCR gels).

We began by testing the stability of the SBW25-Dup-IS genome rearrangement with a stability assay (24). The stability assay involves (i) growing the duplication strain in overnight culture, (ii) plating onto agar, and (iii) examining the size of the resulting colonies. Cells that retain the duplication grow relatively quickly and thus generate large colonies, while cells that have lost the duplication give rise to visibly smaller colonies. On average, 0.91% of colonies derived from overnight SBW25-Dup-IS cultures showed the small colony phenotype, while SBW25Δ*serCGA* (the ancestor of SBW25-Dup-IS) consistently gave rise to colonies of a single size (medians of eight replicates per strain; Fig. 5A; Table S4). These results indicate that, as expected, the duplication carried by strain SBW25-Dup-IS is unstable.

SBW25-Dup-IS duplication fragment loss can, hypothetically, result in several new genomic arrangements. These include (i) loss of the second duplication copy, leaving the new IS element and the first duplication copy (i.e., emergent junction Dup1-IS1 remains), (ii) loss of the first duplication copy, retaining IS and the second duplication (i.e., emergent junction IS2-Dup2 remains), and (iii) clean loss of one duplication copy and the IS element (i.e., neither junction remains). These genomic rearrangements can easily be distinguished from each other using a set of three PCRs. The first PCR, the same as that shown in Fig. 3D, is used to demonstrate duplication loss; a product is only amplified in the presence of the SBW25-Dup-IS duplication. In cases where no product is obtained for PCR1, the second and third PCRs indicate whether the new IS element is retained (PCR2 amplifies junction Dup1-IS1, PCR3 junction IS2-Dup2). If the new IS copy remains, a product should be obtained for either PCR2 or PCR3. Alternatively, if the IS element is lost along with the duplication, none of the three PCRs should amplify a product (Fig. 5B and C).

To test which type(s) of the above genomic rearrangements is occurring in small colonies arising from SBW25-Dup-IS, 16 colonies were isolated from the stability assay (one small and one large colony from each of the eight independent replicates). The three PCRs demonstrate that all eight small colonies had lost both the duplication fragment and the new IS element (while all eight large colonies retain both) (Fig. 5C; see Fig. S1 for gels).

## DISCUSSION

In this work, we have observed multiple examples of IS elements inserted into large-scale genomic rearrangements. Firstly, we have directly observed two such events occurring in bacterial populations evolving in real-time in the laboratory: an IS*481* movement associated with a large duplication in *P. fluorescens* SBW25, and an IS*5* movement associated with a large deletion in *E. coli* C. In addition to occurring together with a large-scale rearrangement, none of the two IS movements presented here generated a TSD. Secondly, we computationally identify a further 14 IS*150* movement events that are associated with large deletions in some *E. coli* B LTEE populations and lack TSDs. However, these 14 IS movement events can all be mechanistically categorized as adjacent deletions. While the transposition process typically generates direct repeats at the insertion site (see the introduction), the existence of (occasional) IS elements with no obvious TSDs has been documented for many years (7). To date, these missing TSDs have been attributed to loss of the TSD at some point after the IS movement (e.g., through recombination between two disparate IS copies, or indels/SNPs or an adjacent deletion) (7). While this may be true in some cases, here we show that *de novo* insertions of IS elements—which have not had time to lose TSDs by subsequent mutation—can also lack TSDs, when they occur in association with large duplications or deletions. We hypothesize that, in these cases, the IS element directly inserts into the double-stranded DNA breaks that occur during the resolution of genomic rearrangements (28). If the IS element does not cut the DNA itself, then the TSDs that result from staggered cuts on the leading and lagging DNA strands are not expected to be introduced.

We are not the first to propose that transposases can take advantage of DNA strand breaks. In the bacterium *Deinococcus radiodurans,* the HUH transposase IS*Dra2* has been observed to transpose during the reassembly of the genome after gamma irradiation (30). Gamma rays destroy the genome but also induce the activity of the HUH transposase, which very quickly inserts into the new DNA breaks. Similarly, Tn*7* (encoding transposase TnpB) has also been suggested to take advantage of DNA breaks for transposition in *E. coli* (31). Together with these earlier studies, our work suggests that insertion into DNA breaks may be a broad phenomenon that occurs not only with HUH transposases (such as that encoded within IS*Dra2*), but also with DDE transposases (such as those encoded within IS*481,* IS*5,* IS*150*, and Tn*7*).

Another example of large rearrangements occurring in conjunction with the insertion of DNA is found in *Helicobacter* and *Neisseria*. In these bacteria, genes encoding restriction-modification systems are inserted into the center of large-scale rearrangements (32). Bioinformatic analyses of closely related strains have shown that large DNA insertions are often accompanied by large intragenomic duplications or deletions (similar to those observed for the IS movements in our work). The authors hypothesize that these insertions are mediated by the restriction modification enzymes attacking target DNA, and subsequent insertion. Alternatively, one could hypothesize that the DNA has been inserted into existing breaks caused by DNA replication errors.

In eukaryotes, double-stranded DNA breaks can be repaired through a non-homologous end-joining mechanism (33). This repair mechanism is often accompanied by the insertion of non-specific DNA into the breakpoint (34). In many cases, retrotransposons are inserted into these DNA breaks (35, 36). While this opportunistic function may provide short-term benefits, it is presumably detrimental to the host in the long term, as it significantly increases the likelihood of future transposition events (37). However, in at least one case, it has been shown that a bacterial group II intron has been domesticated to function as a DNA repair protein in *Pseudomonas aeruginosa* (38).

There are alternative explanations for our DNA break hypothesis. For example, it is possible that the deletion we observed in combination with the IS*5* transposase (in *E. coli* C) is the result of two consecutive mutations: a novel IS insertion, followed by an adjacent deletion. If this is the case, then the quick succession of these two events could have prevented us from observing the new IS copy alone. Similarly, in *P. fluorescens* SBW25 it is possible that the mutation was, in reality, two distinct mutational events: an IS*481* insertion, followed by a duplication event. This duplication event could have been mediated by transposase activity and instead of leading to an adjacent deletion, it led to a duplication of adjacent DNA (potentially by inserting the cointegrate into the same position twice). However, this explanation seems unlikely since we would expect such duplication events to have been observed in at least one of the many previous transposase experiments (e.g., references 15, 16, 19).

Together, our data and analyses suggest a role for some IS elements in generating large-scale deletions and duplications. These mutations are accompanied by a lack of observable TSDs at the insertion site, which suggests that either (i) an IS element inserted in an existing DNA break that is not generated by the endonuclease function of the transposase itself, or (ii) an IS movement event was followed by a deletion or duplication mediated by the IS transposase.

## MATERIALS AND METHODS

### Movement of IS elements in LTEE populations

Raw sequencing reads for three 60,000-generation LTEE populations (Ara+1, Ara-3, Ara-6) were downloaded from the NCBI BioProject database (accession number PRJNA380528) (12). These three populations were chosen because they show high IS activity (37). Each set of raw reads was aligned to the *E. coli* B REL606 reference sequence (NCBI RefSeq NC_012967.1) using breseq (21) on the polymorphism setting (-p). All predicted IS movement events were recorded (Table S1). Next, each predicted IS movement was manually inspected and characterized with respect to (i) association with large genomic rearrangements (defined here as insertions or deletions of >10 bp), and (ii) target site duplications (Table S1). IS movements were then classified into five categories: four categories in which the movements were not associated with other large rearrangements (namely, IS*1*, IS*3,* IS*186*, IS*150*) and one category in which the movements were associated with large deletions (IS*150-*del).

### Growth conditions

Strains used in the laboratory component of this study are listed in Table S5. *E. coli* C strains were grown in lysogeny broth (LB) at 37°C with shaking (~16 hours). *P. fluorescens* SBW25-derived strains were grown in liquid King’s medium B (KB) (39) at 28°C with shaking (~16 hours).

### Genome sequence of Ec-Del-IS

This work provides a detailed bioinformatic and empirical analysis of the previously reported genome sequence of Ec-Del-IS (strain E_coliCRF_phi_GM1_12c1 in reference 22). Using breseq, raw reads were aligned to an updated version of the standard *E. coli* C genome (GenBank CP020543.1). The updated genome differs by the presence of nine insertions and two substitutions (40). A mean coverage of 142-fold per genomic base was obtained. Mutations were identified using a mixture of breseq (21), Geneious (version 2020.1.2), and previously published protocols (23, 24) (see Text S1 and Table S2). Raw sequencing reads, and the updated reference genome, are available on Zenodo.

### Genome sequence of SBW25-Dup-IS

SBW25-Dup-IS was sequenced using Illumina 150 bp paired-end reads at the Max Planck Institute for Evolutionary Biology sequencing facility, Germany. Raw reads (deposited on Zenodo) were aligned to the SBW25 wild-type genome sequence (NCBI RefSeq NC_012660.1 [25]) using breseq (21), giving a mean coverage of 57.6-fold per genomic base. Mutations were identified using a mixture of breseq, Geneious (version 2023.2.1), and previously published protocols (23, 24) (see Text S2 and Table S3).

### PCR and Sanger sequencing

Primers used in this study are listed in Table S5. PCRs were performed under standard conditions for High Performance GoTaq G2 Flexi DNA Polymerase (Promega; M7801), with the addition of 5× combined enhancer solution (41) per reaction. PCR products were visualized on 1% agarose gels (90 V, 30–45 minutes) against a 1 kb DNA ladder (Promega). Sanger sequencing was performed by the sequencing unit at the Max Planck Institute for Evolutionary Biology. Raw gel images and Sanger sequencing traces are provided on Zenodo.

### Stability assay

A stability assay (24) was used to test for duplication fragment loss from SBW25-Dup-IS in overnight culture. The SBW25-Dup-IS duplication confers a growth advantage; cells carrying the fragment give rise to large colonies, while those that have lost the duplication generate smaller colonies. Eight replicate colonies of SBW25-Dup-IS and SBW25Δ*serCGA* (a control strain from which only small colonies are expected) were grown to stationary phase in overnight culture (28°C, shaking). Each of the 16 cultures was dilution plated on 1.5% KB agar, and plates were incubated at room temperature (~20°C) for 48 hours. The numbers and sizes of the resulting colonies were recorded (minimum 146 colonies per replicate; Table S4). Per culture, an instability measure—the proportion of colonies differing in size from the original genotype (SBW25-Dup-IS or SBW25Δ*serCGA*)—was calculated. A Wilcoxon rank sum test was used to test for a difference in the median instability measure of SBW25-Dup-IS and SBW25Δ*serCGA* (Fig. 5A).

## Data Availability

All raw data, including NGS data and associated reference sequences, Sanger sequencing, traces, and gel images, are available on Zenodo (doi:10.5281/zenodo.11402078).

## SUPPLEMENTAL MATERIAL

Text S1 mbio.03052-24-s0001.pdf Genome sequence of Ec-Del-IS.

Text S2 mbio.03052-24-s0002.pdf Genome sequence of SBW25-Dup-IS.

Figure S1 mbio.03052-24-s0003.eps PCR results demonstrating the loss of the duplication fragment and the associated new IS*481* copy in small colony isolates.

Supplemental legends mbio.03052-24-s0004.docx Legends for supplemental figures and tables.

Table S1 mbio.03052-24-s0005.xlsx Characteristics of the IS element movement events detected by breseq in three LTEE populations at 60,000 generations (Ara+1, Ara-3, Ara-6).

Table S2 mbio.03052-24-s0006.xlsx Ec-Del-IS predicted mutation details.

Table S3 mbio.03052-24-s0007.xlsx SBW25-Dup-IS predicted mutation details.

Table S4 mbio.03052-24-s0008.xlsx Raw colony counts for the SBW25-Dup-IS stability test.

Table S5 mbio.03052-24-s0009.xlsx Details of genotypes, PCR primers, and emergent junction sequences used in this study.

ASM does not own the copyrights to Supplemental Material that may be linked to, or accessed through, an article. The authors have granted ASM a non-exclusive, world-wide license to publish the Supplemental Material files. Please contact the corresponding author directly for reuse.
