# Supplementary material for: A more significant role for insertion sequences in large-scale rearrangements in bacterial genomes
Source: mBio. 2024 Dec 5;16(1):e03052-24. doi: 10.1128/mbio.03052-24 (PMC11708052; doi:10.1128/mbio.03052-24)
Supplement: Text S1 — Genome sequence of Ec-Del-IS. [file mbio.03052-24-s0001.pdf]

Supplementary Text S1
The genome sequence of Ec-Del-IS

1.1 The genomic rearrangement in Ec-Del-IS

- A 62,007-bp deletion (3,612,369 – 3,674,375 inclusive), plus a new IS*5*/IS*1182* element copy in place of the deleted fragment.
- There are 3 identical copies of IS element in Ec-Del-IS, and two identical (complete) copies in the wildtype *E. coli* C genome.

>Ec-Del-IS
TGCCGGACCGCCGACC**ACATACACGCCACAGGAGG**CGTTATCCGCCACGGTATCAACAAATTTGATCGACCAGTC
GCGGATACGGCTGCCGACCACTTCCAGCGCGGGAAGTACCCACTCTATGGCGTTGTAAAGCTCGTCAAAAGGGCG
TTGTTGCTCAAATCAGGGAACTATCCCGCAGTTTGGTGATCTGATCTCTTAGGTTCACCACCGGGAGCGCCAG*AT*
GGGCAAGTCCAGGCACAAGATCAGCAACTGGAAACAGTACAACCAAGCCTTGGTGCAACGTGGTTCGTTGACCGT
*CTGGATGGATGAGCAAGCCATCCAGCAGTGGCATTGCCAAACCCATCATGGCCGCCGAGGG****CGAGGCTTCCATTA***
CAGC*GACTCCGCCATTGAAACCGCGCTGATGCTCAAGGCGGTATTCAAGCTACCGCTGCGTGCCCTGGAAGGCTT*
CATCAACTCGCTGTTCAAGCTGATGAAGGTGCCCTTACAGTCGCCGGACTACAGCTGTATCAGCAAGCGCGCCAA
GACGGTCGAAATCAAGTACCGCCTACCGAGCCATGGCCCAGTGGCTCACCTGGTCATCGATGCCACCGGACTCAA
GGTCTATGGCGAAGGCGAATGGAAAATTCGCAAGCACGGCAAGGAGAAACGCCGCGTCTGGCGCAAGCTTCACCT
*GGCCGTAGATGCGG****CGACCCATGCCATCGTTG****CAGCCGAAGTCAGCCTGGAAACGGTGGGCGATAACGAGGTGTT*
GCCCACGCTGCTCAACCCCTTGCGCCGCAAGATAGAACAAGTCAGCGCCGATGGTGCCTATGACACCAGAGCCTG
TTATGCCCTACTGCAAAAGAAGGGCGCCAAGGCCACCATACCGCCGAGAAAAAATGCGGCATTCTGGAAGACAGG
*CCATCCACGCAACGAGGCGGTTGCCGCGCTCAAGGCAGGAGAACTGGAGCAATGGAAAAAGG****ACTCTGGCTATCA***
TCAGCGC*TCGATAGCTGAGACCGCCATGTATCGCTTCAAGCAGCTCATCGGTCCGAAACTGAGTCTGCGGAGCTA*
CAACGCCCAGGTGGGTGAGATCCTGGCCGGGGTGAAAGTCATGAACAAGGTCATAGGGCTTGGTATGCCTATTCG
*GCAGGCTGTCAACTAA*GCCCGCCCGGCGGGTTGGG**GAGCGACCATCCAACTTTG**GATTTGATCAACAAGGCCTTT
AGACACCCAAAAAGAGAACCGCCCCAAATGTGTGACATATCTGGATCTATTTGTGCAGTTTTACATAACATCATA
CACCGCTCATGTATTTCTCCGCAGTCACTATCGTATGCTGTTTGGCAGGTTAATAAATATCCAGATGATTCCCCG
TTACAAGACGAAATTGTCGATTCCATCTTACTTTTAAGTAATGCGGATAAGTTTTGCATTGACAATGCTGGAGCC
ATAACAGATAGCCCCAT**CGGATCAATGTTCATAACCGGACTC**AACGGATAAATGTATAAATTCCATCCCCCCTCC
A

BLUE end of gene 1 (*B6N50_18465*, encoding a 2-keto-4-pentenoate hydratase); this is the region where the deletion fragment starts (i.e., Junction 1: Del1-IS1)
BLACK IS element sequence (1,051-bp)
ITALICS transposase gene (918-bp)
GREEN single nucleotide from gene 2 (*B6N50_18845*, encoding a hypothetical protein) at Junction 2 (IS2-Del2)
GREEN intergenic region at end of deletion (i.e., Junction 2: IS2-Del2)
PURPLE gene 3 (*B6N50_18850*, encoding a hypothetical protein), after the intergenic region downstream of junction 2 (IS2-Del2)

30-bp Junction 1 sequence: "Del1-IS1": AAAGCTCGTCAAAAGGGCGTTGTTGCTCAA (grey = side 1, turquoise = side 2)
30-bp Junction 2 sequence: "IS2-Del2": TTGATCAACAAGGCCTTTAGACACCCAAAA (grey = side 1, green / turquoise = side 2)

1.2 PCR based evidence for Ec-Del-IS genome rearrangement

1.2.1 PCR to amplify the entire rearrangement region

DelIS_LP_F: **ACATACACGCCACAGGAGG**
DelIS_LP_R: **GAGTCCGGTTATGAACATTGATCCG**

Expected product sizes:
wild type: ~62 kb (i.e., too large to amplify here)
Ec-Del-IS: 1,451 bp

Deletion without new IS element copy: ~400 bp (as far as we know, this doesn't exist)

Additional primers to sequence through PCR product:
DelIS_LP_seq_F1: **CGAGGCTTCCATTACAGC**
DelIS_LP_seq_R1: **GCTGTAATGGAAGCCTCG** (reverse complement of DelIS_LP_seq_F1)
DelIS_LP_seq_F2: **CGACCCATGCCATCGTTG**
DelIS_LP_seq_F3: **ACTCTGGCTATCATCAGCGC**
DelIS_LP_seq_F4: **GAGCGACCATCCAACTTTG**

1.2.2 PCRs for amplifying each emergent junction

Junction 1 (Del1-IS1)
DelIS_LP_F: **ACATACACGCCACAGGAGG**
DelIS_LP_seq_R1: **GCTGTAATGGAAGCCTCG**

Expected product sizes:
wild type: no product expected
Ec-Del-IS: 363 bp
Deletion without new IS element copy: no product expected

Junction 2 (IS2-Del2)
DelIS_LP_seq_F4: **GAGCGACCATCCAACTTTG**
DelIS_LP_R: **GAGTCCGGTTATGAACATTGATCCG**

Expected product sizes:
wild type: no product expected
Ec-Del-IS: 307 bp
Deletion without new IS element copy: no product expected

1.2.3 Probe PCR for amplifying a wild-type-specific product

DelIS_LP_F: **ACATACACGCCACAGGAGG**
DelIS_WT_LP_R **GAAGCCGAAATTGCGCTGGTG** (binds in WT genome but not Ec-Del-IS)

Expected product sizes:
wild type: 182 bp
Ec-Del-IS: no product expected
Deletion without new IS element copy: no product expected

>Ecoli_C_WT_genome
…**ACATACACGCCACAGGAGG**CGTTATCCGCCACGGTATCAACAAATTTGATCGACCAGTCGCGGATACGGCTGCC
GACCACTTCCAGCGCGGGAAGTACCCACTCTATGGCGTTGTAAAGCTCGTCAAAAGTGGTGTCGGTATTGGGCAG
ATCGCGGTTCAG**CACCAGCGCAATTTCGGCTTC**AATACGCGGTTGCAGCACACGGGAAAACGGGATGGTTTCGTT
ATCGCCATAACACATATCAGCAAACAGCGTACCGAAGTCCGGTTGATCGACACCCAGCTGTTGCTGCACTTTCGG
GTGCGTCAG…

Yellow deleted in Ec-Del-IS
