# Supplementary material for: A more significant role for insertion sequences in large-scale rearrangements in bacterial genomes
Source: mBio. 2024 Dec 5;16(1):e03052-24. doi: 10.1128/mbio.03052-24 (PMC11708052; doi:10.1128/mbio.03052-24)
Supplement: Supplemental legends — Legends for supplemental figures and tables. [file mbio.03052-24-s0004.docx]

**Legends for Supplementary Figures and Tables**

**Supplementary Figure S1. PCR results demonstrating the loss of the duplication fragment and the associated new IS*481* copy in all small colony isolates.** Agarose gel of small and large colony PCR products amplified with (**A**) external primers targeting both emergent junctions and the new IS*481* copy, (**B**) primers targeting junction Dup1-IS1, and (**C**) junction IS2-Dup2. Markers are 1 kb DNA ladders from Promega.

**Supplementary Table S1. Characteristics of the IS element movement events detected by *breseq* in three LTEE populations at 60,000 generations (Ara+1, Ara-3, Ara-6).** IS movement events were sorted into categories, based on (i) the identity of the IS element (IS*1*, IS*3*, IS*150*, IS*186*), and (ii) whether or not the movement was associated with a deletion of >10 bp (“IS-del”). Target site duplications (TSDs) were analysed for each IS movement.

**Supplementary Table S2. Ec-Del-IS predicted mutation details.** The first tab provides details of the mutations predicted in the Ec-Del-IS genome by *breseq*. The second tab contains a list of the 152 genes predicted to fall within the 62,007 bp deleted region.

**Supplementary Table S3. SBW25-Dup-IS predicted mutation details.** The first tab provides details of the mutations predicted in the SBW25-Dup-IS genome by *breseq* and/or Geneious. The second tab contains a list of the 63 genes predicted to fall within the 59,443 bp duplicated region.

**Supplementary Table S4. Raw colony counts for the SBW25-Dup-IS stability test.**

**Supplementary Table S5. Details of genotypes, PCR primers, and emergent junction sequences used in this study.**
